# Analysis of Risk Factors for Distant Metastasis of Pancreatic Ductal Adenocarcinoma without Regional Lymph Node Metastasis and a Nomogram Prediction Model for Survival

**DOI:** 10.1155/2023/2916974

**Published:** 2023-02-21

**Authors:** Jinsheng Huang, Xujia Li, Qi Jiang, Huijuan Qiu, Yuming Rong, Bokang Cui, Guifang Guo

**Affiliations:** ^1^VIP Department, Sun Yat-sen University Cancer Center, 651 Dongfeng Road East, Guangzhou 510060, China; ^2^State Key Laboratory of Oncology in South China, Sun Yat-sen University Cancer Center, 651 Dongfeng Road East, Guangzhou 510060, China; ^3^Collaborative Innovation Center for Cancer Medicine, Sun Yat-sen University Cancer Center, 651 Dongfeng Road East, Guangzhou 510060, China; ^4^Department of Pancreaticobiliary Surgery, Sun Yat-sen University Cancer Center, 651 Dongfeng Road East, Guangzhou 510060, China

## Abstract

**Background:**

Negative regional lymph nodes do not indicate a lack of distant metastasis. A considerable number of patients with negative regional lymph node pancreatic cancer will skip the step of regional lymph node metastasis and directly develop distant metastasis.

**Methods:**

We retrospectively analyzed the clinicopathological characteristics of patients with negative regional lymph node pancreatic cancer and distant metastasis in the Surveillance, Epidemiology, and End Results database from 2010 to 2015. Multivariate logistic analysis and Cox analysis were used to determine the independent risk factors that promoted distant metastasis and the 1-, 2-, and 3-year cancer-specific survival in this subgroup.

**Results:**

Sex, age, pathological grade, surgery, radiotherapy, race, tumor location, and tumor size were significantly correlated with distant metastasis (*P* < 0.05). Among these factors, pathological grade II and above, tumor site other than the pancreatic head, and tumor size >40 mm were independent risk factors for distant metastasis; age ≥60 years, tumor size ≤21 mm, surgery, and radiation were protective factors against distant metastasis. Age, pathological grade, surgery, chemotherapy, and metastasis site were identified as predictors of survival. Among them, age ≥40 years, pathological grade II and above, and multiple distant metastasis were considered independent risk factors for cancer-specific survival. Surgery and chemotherapy were considered protective factors for cancer-specific survival. The prediction performance of the nomogram was significantly better than that of the traditional American Joint Committee on Cancer tumor, node, metastasis staging system. We also established an online dynamic nomogram calculator, which can predict the survival rate of patients at different follow-up time points.

**Conclusion:**

Pathological grade, tumor location, and tumor size were independent risk factors for distant metastasis in pancreatic ductal adenocarcinoma with negative regional lymph nodes. Older age, smaller tumor size, surgery, and radiotherapy were protective factors against distant metastasis. A new nomogram that was constructed could effectively predict cancer-specific survival in pancreatic ductal adenocarcinoma with negative regional lymph nodes and distant metastasis. Furthermore, an online dynamic nomogram calculator was established.

## 1. Introduction

Invasion and metastasis are important biological characteristics of malignant tumors and are also the main reasons for the difficulty and failure of tumor treatment [1]. Therefore, invasion and metastasis have become an urgent problem in current biomedicine and tumor research that needs to be resolved. Pancreatic ductal adenocarcinoma (PDAC) is a malignant tumor with strong invasive potential that is highly prone to metastasis [2]. PDAC is the fourth leading cause of cancer death, as the 5-year survival rate is less than 5% [3]. Due to the occult onset of PDAC and atypical early symptoms, most patients have already developed local progression or metastasis at the time of diagnosis, and surgical resection is possible in only 15–20% of patients [4], and most patients still experience recurrence and metastasis after surgery [5]. Without treatment, the median survival time of patients with metastatic PDAC is only 3 months [6]. In recent years, based on the development of early detection methods and the diversification of treatment models, the overall survival rate of patients with pancreatic cancer has improved to some extent, but the long-term survival of these patients is still not optimistic, especially for those with distant metastasis [7].

The status of regional lymph nodes plays an important role in predicting the biological invasiveness and metastatic tendency of PDAC. The regional lymph node status is a key component of the tumor staging system [8] and is an important factor that affects prognosis [9]. Patients with regional lymph node metastasis are more likely to have distant metastasis, and their prognosis is significantly worse than that of patients with negative regional lymph nodes (*N*0) [10]. PDAC patients with regional lymph node metastasis often require more treatment and closer monitoring [11]. *N*0 is considered a good prognostic indicator. When exploring the risk factors for distant metastasis, *N*0 pancreatic cancer patients are often assigned to the low-risk group (the control group) in studies [12]. However, surprisingly, during the literature review, we found that a considerable number of patients with *N*0 pancreatic cancer still had distant metastasis [13, 14]. This finding suggests that clinicians may underestimate the risk of recurrence and metastasis in *N*0 patients and that previous treatment and monitoring strategies may not be optimal. Inadequate prediction of the risk of metastasis may lead to insufficient adjuvant therapy in *N*0 pancreatic cancer patients and an increased risk of recurrence and metastasis.

This study aimed to explore the potential risk factors that promote the development of distant metastasis in PDAC with *N*0 to predict the cancer-specific survival (CSS) rate of this patient subgroup and to establish a predictive model to evaluate the prognosis of PDAC patients with *N*0 and distant metastasis. The prognosis of metastatic PDAC is extremely poor, and the quality of life and treatment effects in patients are often unsatisfactory. Therefore, early prediction of the risk of distant metastasis is vital to the treatment and prognosis of PDAC. For high-risk subgroups of patients, more active treatment and monitoring may be the key to improving the survival rate.

## 2. Materials and Methods

### 2.1. Data Source

This was an observational retrospective cohort study that collected data of pancreatic cancer patients (*n* = 71,359) in the Surveillance, Epidemiology, and End Results (SEER) database derived from 18 cancer registries between 2010 and 2015. Among them, 21,547 patients had lymph node metastasis and 49,812 patients did not have lymph node metastasis (as the official coding manual described, the diagnosis of *N*0 pancreatic cancer patients was not only dependent on surgical resection, but a majority of patients could be diagnosed with needle aspiration (cytology) or core biopsy (tissue)). According to the inclusion and exclusion criteria, 22,976 patients without lymph node metastasis were screened and assessed. Patients who were lost to follow-up and those with incomplete medical records were excluded (*n* = 18,305). Finally, 4491 patients with *N*0 pancreatic cancer (confirmed by histology), including those with *N*0 and no-distant metastasis (*n* = 2953) and those with *N*0 and distant metastasis (*n* = 1538) were included in the study. The collection and analysis of medical records were performed independently by two researchers to reduce selection bias. The report of this study follows the guidelines of the strengthening the reporting of observational studies in epidemiology (STROBE) statement [15].

**Figure 1:**
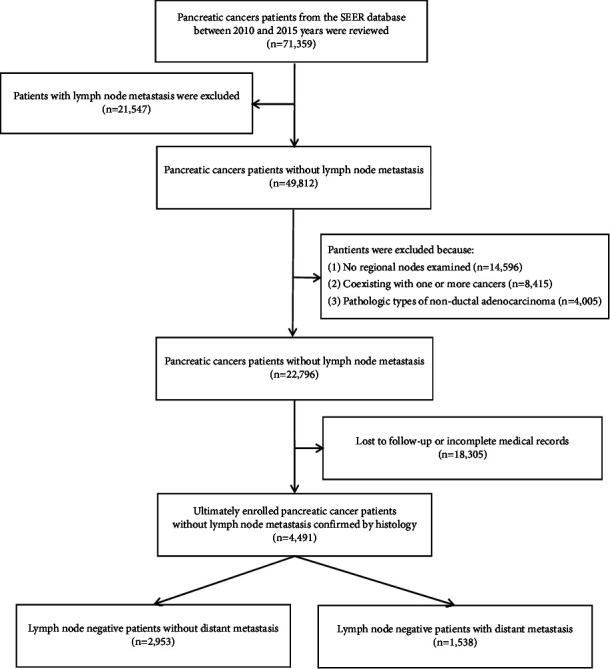
The patients' selection processing.

The inclusion criteria included (1) histologically confirmed PDAC; (2) the pancreas as the primary lesion site; and (3) complete demographic information, such as age, sex, race, stage, treatment method, follow-up, and prognosis. The exclusion criteria included the following: (1) no regional nodes examined; (2) coexistence of PDAC and at least one other cancer; (3) diagnosis of nonductal adenocarcinoma pathologic types; and (4) patients who were lost to follow-up and those with incomplete medical records. The screening process is illustrated in Figure 1.

### 2.2. Variable Evaluation and the Definition

According to the sample size requirement for establishing the multivariate linear regression equation, the sample size in this study should be at least 10 times the number of independent variables in the equation. Therefore, after excluding unqualified cases, 4491 patients in PDAC with *N*0 participated in this study. The risk factors that promote the development of distant metastasis in *N*0 pancreatic cancer were investigated through the random assignment. In addition, to predict the CSS at 1, 2, and 3 years, patients in PDAC with *N*0 and distant metastasis from 2010–2015 were randomly divided into the training cohort (*n* = 1083) and the validation cohort (*n* = 455) at a ratio of 7 : 3.

Variables were selected based on the correlation between the variables and the purpose of the study. The following clinical and pathological characteristics were collected and converted into categorical variables: age (20–39 years, 40–59 years, 60–79 years, and ≥80 years), race (white, black, Asian or Pacific Islander, and American Indian/Alaska Native), pathological grade (grade I, well differentiated; grade II, moderately differentiated; and grade III/IV, poorly differentiated and undifferentiated), location (the pancreatic head, pancreatic body, pancreatic tail, and other parts), tumor size (*T*1, 0–20 mm; *T*2, 21–40 mm; and *T*3, >40 mm), and metastasis (the liver, the lung, other sites, and multiple sites).

### 2.3. Statistical Analysis

The primary endpoint of this study was distant metastasis and the 1-, 2-, and 3-year CSS. Using R package “bestglm” to perform the univariate and multivariate logistic analyses, the potential independent clinical risk factors for the development of distant metastasis in *N*0 patients were identified. Using R package “survival” to perform the univariate and multivariate Cox regression analyses which were used to investigate the factors that affect the prognosis in terms of CSS at 1, 2, and 3 years. When performing reverse stepwise selection, we used a two-tailed *P* value <0.05 as the criterion for variable deletion. Based on a multivariate Cox regression analysis, use R package “rms” to create nomogram curves and calibration curves, and use R package “survminer” to perform Kaplan–Meier analyses. The recognition ability of this nomogram was evaluated by calculating Harrell's *C*-index which is created by R package “rms” and plotting the time-dependent receiver operating characteristic (ROC) curve which is drawn by R package “timeROC.” The DCA curve which is drawn by R package “ggDCA” was used to assess the utility of nomograms for decision making. Finally, the nomogram based on the aforementioned Cox regression model was converted into an online web calculator (dynamic nomogram analysis) created by R package “DynNom” that is convenient for clinical applications. All analyses were performed using R software (version 4.0.2).

## 3. Results

### 3.1. Clinicopathological Characteristics of Lymph Node-Negative Patients

Overall, between 2010 and 2015, 4491 patients in PDAC with *N*0 and a median age at diagnosis of 67 years (in the range of 26–85 years) were enrolled in this study. Among the 4491 *N*0 patients, 1538 (34.24%) patients also had distant metastasis, including 1083 cases in the training cohort and 455 cases in the validation cohort. Clinicopathological characteristics of both cohorts are listed, respectively, in Table 1 and the baseline of all characteristics were balanced. Most patients accepted chemotherapy, while the minorities were given surgery and radiotherapy.

### 3.2. Univariate and Multivariate Logistic Analyses of Risk Factors for Distant Metastasis

We performed a logistic regression analysis to investigate the potential clinical risk factors of distant metastasis in PDAC with *N*0 patients. In the univariate logistic analysis, sex (*P*=0.002), age (*P* < 0.001), pathological grade (*P* < 0.001), surgery (*P* < 0.001), radiation (*P* < 0.001), race (*P*=0.005), location (*P* < 0.001), and tumor size (*P* < 0.001) were significantly correlated with distant metastasis. Therefore, we combined these eight clinicopathological factors into a multivariate logistic analysis to predict the tumor size (*P* < 0.001) that were significantly correlated with the distant metastasis risk. We performed a logistic regression analysis to investigate the potential clinical risk factors of tumor size (*P* < 0.001) that were significantly correlated with distant metastasis in PDAC with *N*0 patients, and this combination showed good predictive power. The area under the ROC curve (AUC) was 0.87 (95% CI: 0.86–0.88) (Figure S1). The following were independent risk factors for distant metastasis: age (40–59 years: OR = 1.01, 95% CI: 0.39–2.65, *P* < 0.001), pathological grade (grade II: OR = 1.77, 95% CI: 1.35–2.32; grade III/IV: OR = 2.05, 95% CI: 1.57–2.68; *P* < 0.001), location (body: OR = 2.25, 95% CI: 1.79–2.84; tail: OR = 4.78, 95% CI: 3.71–6.19; other: OR = 1.87, 95% CI: 1.52–2.31; *P* < 0.001), and tumor size (>40 mm: OR = 1.24, 95% CI: 0.9–1.71, *P*=0.006). Chemotherapy (*P*=0.941) was not statistically significant as a risk factor. In contrast, older age (60–79 years: OR = 0.8, 95% CI: 0.32–2.1; ≥80 years: OR = 0.36, 95% CI: 0.14–0.94; *P* < 0.001), smaller tumor size (21–40 mm: OR = 0.95, 95% CI: 0.69–1.3, *P*=0.006), surgery (OR = 0.05, 95% CI: 0.03–0.06, *P* < 0.001), and radiation (OR = 0.15, 95% CI: 0.12–0.19, *P* < 0.001) were protective factors for distant metastasis. Sex (*P*=0.528) and race (*P*=0.220) were also not statistically significant (Table 2).

### 3.3. Univariate and Multivariate Cox Analysis of Risk Factors for CSS

We performed a survival analysis of all PDCA patients with *N*0 and distant metastasis (Figure S2A). Meanwhile, the training cohort and validation cohort were also analyzed separately (Figures S2B and S2C). The results show that the median CSS of the overall cohort, training cohort, and validation cohort were 5.0, 5.0, and 4.0 months, respectively. The 1-, 3-, and 5-year CSS rates of the overall cohort, training cohort, and validation cohort were 20.85%, 21.16%, and 20.11%; 2.99%, 3.17%, and 2.57%; 1.26%, 1.39%, and 0.76%, respectively.

To clarify the independent risk factors for the 1-, 2-, and 3-year CSS in PDAC with *N*0 and distant metastasis patients, we further included the significant factors of the univariate Cox regression analysis in the multivariate Cox regression analysis. In the univariate Cox regression analysis, age (*P* < 0.001), pathological grade (*P* < 0.001), surgery (*P* < 0.001), chemotherapy (*P* < 0.001), and metastatic site were identified as predictors. However, sex (*P* = 0.672), radiation (*P* = 0.05), race (*P* = 0.102), location (*P* = 0.2), and tumor size (*P* = 0.192) were not statistically significant. Furthermore, the multivariate Cox regression analysis showed that age (40 to 59 years: HR = 2.23, 95% CI: 0.99–5.02; 60 to 79 years: HR = 2.37, 95% CI: 1.06–5.32; ≥80 years: HR = 3.69, 95% CI: 1.62–8.43; *P* < 0.001), pathological grade (grade II: HR = 1.5, 95% CI: 1.18–1.91 and grade III/IV: HR = 2.02, 95% CI: 1.58–2.57; *P* < 0.001), and multiple metastatic sites (HR = 1.31, 95% CI: 1.07–1.59, *P* < 0.001) were considered independent risk factors for CSS. Surgery (HR = 0.45, 95% CI: 0.34–0.59, *P* < 0.001) and chemotherapy (HR = 0.45, 95% CI: 0.39–0.52, *P* < 0.001) were considered protective factors for CSS. Compared with multiple metastases, pulmonary metastasis (HR = 0.81, 95% CI: 0.62–1.05, *P* < 0.001) and metastases at other sites (HR = 0.8, 95% CI: 0.68–0.95, *P* < 0.001) had longer CSS (Table 3).

**Figure 2:**
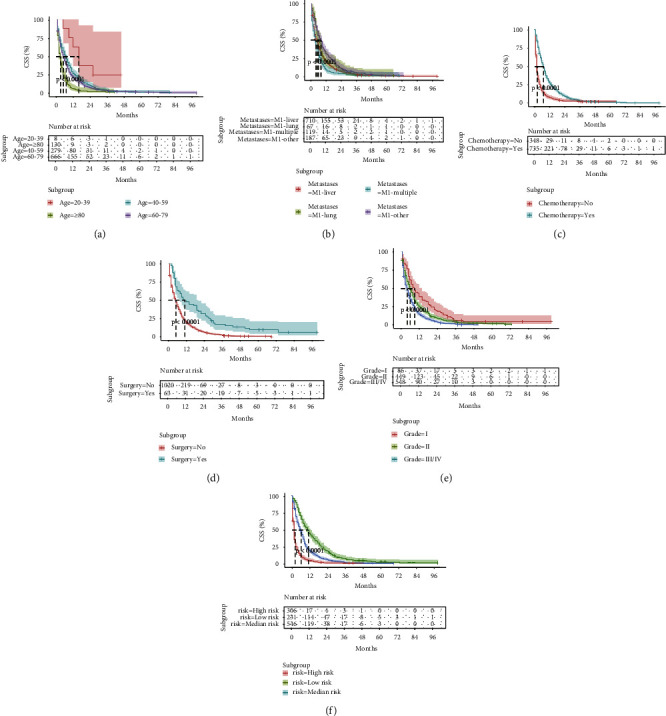
The Kaplan–Meier survival curves for predicting the CSS of lymph-node-negative PDAC with distant metastasis. (a) Kaplan–Meier curves in age groups. (b) Kaplan–Meier curves in the metastases groups. (c) Kaplan–Meier curves in the chemotherapy groups. (d) Kaplan–Meier curves in the surgery groups. (e) Kaplan–Meier curves in the grade groups. (f) Kaplan–Meier curves in the risk groups. *P* < 0.05 was considered statistically significant. CSS: cancer-specific survival; PDAC: pancreatic ductal adenocarcinoma.

To accurately estimate the probability of survival and the cumulative risk of patients under the effect of different variables, Kaplan–Meier survival curves were plotted using five predictors (*P* ≤ 0.05) from the Cox proportional risk model. The results were as follows: The median CSS of patients aged 20–39 years, 40–59 years, 60–79 years, and ≥80 years were 16.0, 7.0, 5.0, and 3.0 months, respectively, and the 1-, 2-, and 3-year CSS rates of these patients were 62.5%, 26.7%, 21.3%, and 6.2%; 25.0%, 10.5%, 7.2%, and 2.3%; and NR (not reached), 3.9%, 3.0%, and 1.5%, respectively. The median CSS rates of patients with pathological grade I, grade II, and grade III/IV disease were 9.0, 6.0, and 4.0 months, respectively, and the 1-, 2-, and 3-year CSS rates of these patients were 38.9%, 25.2%, and 15.1%; 18.9%, 9.6%, and 4.4%; and 6.5%, 4.7%, and 1.4%, respectively. The median CSS of patients who underwent surgery and those who did not were 11.0 and 5.0 months, respectively, and the 1-, 2-, and 3-year CSS of these patients were 47.6% and 19.5%; 28.6% and 6.2%; and 15.4% and 2.3%, respectively. The median CSS of patients who received chemotherapy and those who did not was 7.0 and 2.0 months, respectively, and the 1-, 2-, and 3-year CSS rates of these patients were 27.6% and 7.5%; 9.7% and 3.1%; and 3.5% and 2.5%, respectively. The median CSS rates of patients with metastatic sites in the liver, lung, other sites, and multiple sites were 5.0, 6.0, 8.0, and 4.0 months, respectively, and the 1-, 2-, and 3-year CSS rates of these patients were 20.4%, 22.4%, 31.0%, and 9.2%; 6.9%, 10.5%, 11.8%, and 3.4%; and 2.9%, 1.5%, 5.4%, and NR, respectively. These results suggest that patients aged ≥80 years, those with pathological grade III/IV disease, those who were not treated with surgery and chemotherapy, and those with multiple distant metastasis have poor 1-, 2-, and 3-year CSS (Table S1, Figures 2(a)–2(e)).

### 3.4. Construction and Validation of the Nomogram Prediction Model

**Figure 3:**
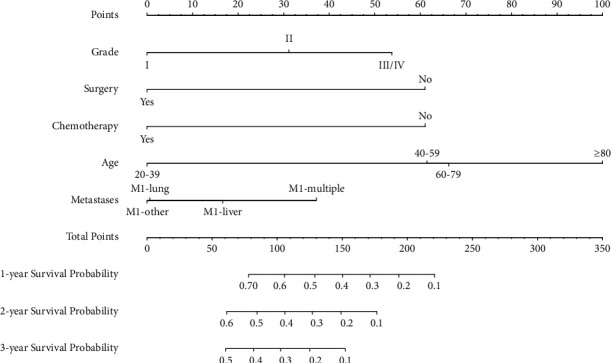
Nomogram for predicting the 1-, 2-, and 3-year CSS in lymph-node-negative PDAC with distant metastasis. The points for each variable can be estimated by plotting a vertical line upwards from the patient's variable values to the top axis marked as “points.” A vertical line is drawn downwards from the sum of all variable values on the “total points” axis to calculate 1-, 2-, and 3-year survival probability; CSS: cancer-specific survival; PDAC: pancreatic ductal adenocarcinoma; Grade: I, well differentiated; II, moderately differentiated; III/IV, poorly differentiated and undifferentiated; *M*1: distant metastases; other: metastasis to other sites except the liver, the lung and multiple lesions.

**Figure 4:**
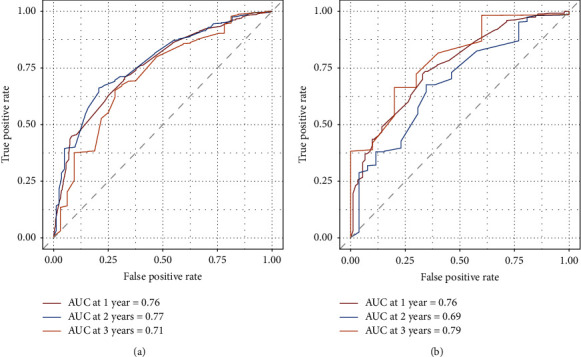
The receiver operating characteristics (ROC) curve and area under the ROC curve (AUC) for 1-, 2-, and 3-year CSS in lymph-node-negative PDAC with distant metastasis. Different colours represent different curves, where red, blue, and orange represent the nomogram model for 1-, 2-, and 3-year CSS, respectively. (a) In the training cohort, the AUC values for the 1-, 2-, and 3-year CSS nomogram were 0.76, 0.77, and 0.71, respectively. (b) In the validation cohort, the AUC values for the 1-, 2-, and 3-year CSS nomogram were 0.76, 0.69, and 0.79, respectively. ROC: receiver operating characteristic; AUC: area under the curve; CSS: cancer-specific survival; PDAC: pancreatic ductal adenocarcinoma.

**Figure 5:**
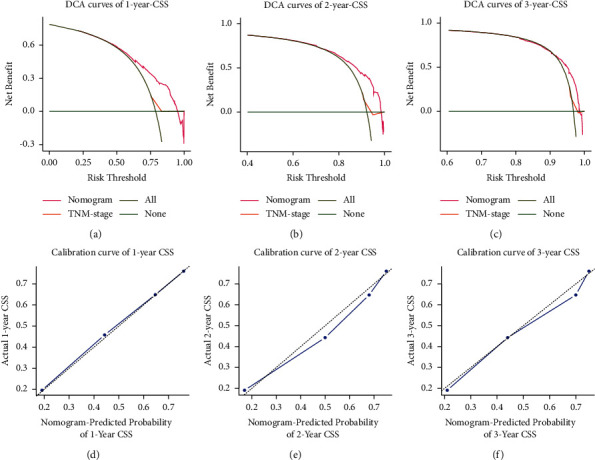
(a)–(c) DCA curves of the nomogram and the TNM staging system. (a) DCA curves for 1-year CSS in the training cohort; (b) DCA curves for 2-year CSS in the validation cohort; and (c) DCA curves for 3-year CSS in the validation cohort. The *y*-axis represents the net benefit, and the *x*-axis represents the corresponding risk threshold. The solid olive line represents all patients who died during the follow-up period. The green solid line represents no patient deaths during the follow-up period. The solid red line represents the net benefit of the nomogram at different risk thresholds. The orange solid line represents the net benefit of TNM staging at different risk thresholds. (d)–(f) Calibration curves for evaluating the accuracy of the nomogram. (d) 1-year CSS in lymph-node-negative PDAC with distant metastasis, (e) 2-year CSS in lymph-node-negative PDAC with distant metastasis, and (f) 3-year CSS in lymph-node-negative PDAC with distant metastasis. The solid blue line represents the performance of the nomogram, of which the closer fit to the dotted gray line represents the better prediction of the nomogram we constructed. CSS: cancer-specific survival; PDAC: pancreatic ductal adenocarcinoma; DCA: decision curve analysis; TNM: tumor node metastasis.

The prediction model was presented in the form of a nomogram. Based on multivariate Cox regression analysis, five predictive indicators (age, pathological grade, surgery, chemotherapy, and metastatic site) were selected for model development. A nomogram data graph to predict the 1-, 2-, and 3-year CSS in PDAC with *N*0 and distant metastasis patients was constructed using the training cohort (Figure 3). Each factor represented a score on the integral scale, and the specific values of individual patients were summed to calculate the total score (see Table 4 for the specific values of each variable). The concordance index (*C*-index), ROC curve, calibration curve, and decision curve analysis (DCA) were used to evaluate the efficacy and clinical application value of the nomogram. The results showed that the *C*-index of the training cohort and the validation cohort nomograms were 0.698 (±0.009) and 0.694 (±0.014), respectively (Table S2), which suggests good discrimination ability. The AUCs of the 1-, 2-, and 3-year CSS for the training cohort were 0.76 (95% CI: 0.72–0.79), 0.77 (95% CI: 0.72–0.82), and 0.71 (95% CI: 0.62–0.81), respectively, which suggests good predictive power (Figure 4(a), Table S2). The subsequently established nomogram model was validated with the internal validation cohort of 455 patients. The results of the validation cohort also showed good discrimination ability. The predicted AUCs for the 1-, 2-, and 3-year CSS were 0.76 (95% CI: 0.70–0.81), 0.69 (95% CI: 0.58–0.79), and 0.79 (95% CI: 0.66–0.92), respectively (Figure 4(b), Table S2). DCA was used to evaluate the utility of the new model in predicting prognosis. We compared the nomogram model with the 8th edition of the AJCC TNM staging system. The analysis showed that the DCA curves for the 1-, 2-, and 3-year CSS rates of the training cohort were higher than the other two reference lines within the larger risk threshold interval, which suggests better predictive efficacy of the nomogram and significantly better predictive ability than the conventional AJCC TNM staging system (Figures 5(a)–5(c)). After construction of the nomogram model, its accuracy was evaluated and verified. The results showed that the calibration curves of the 1-, 2-, and 3-year CSS rates of the training cohort were highly coincident with the actual 1-, 2-, and 3-year CSS curves; this indicates that the prediction results were in good agreement with the actual results, which shows good consistency (Figures 5(d)–5(f)). Similarly, the calibration curves of the 1-, 2-, and 3-year CSS rates in the validation cohort were also highly consistent with the actual 1-, 2-, and 3-year CSS curves (Figure S3). In addition, the patients were divided into low- (*n* = 231), moderate- (*n* = 546), and high-risk groups (*n* = 306) according to the upper and lower quartiles. The median CSS rates of the low-, moderate-, and high-risk groups were 11.0, 6.0, and 2.0, respectively. The 1-, 2-, and 3-year CSS rates for the low-, moderate-, and high-risk groups were 45.0%, 19.9%, and 5.3%; 19.1%, 6.3%, and 1.5%; and 6.8%, 2.2%, and 1.1%, respectively (Figure 2(f)), which suggests that the lower the risk group is, the higher the survival rate is.

### 3.5. Dynamic Nomogram Calculator

**Figure 6:**
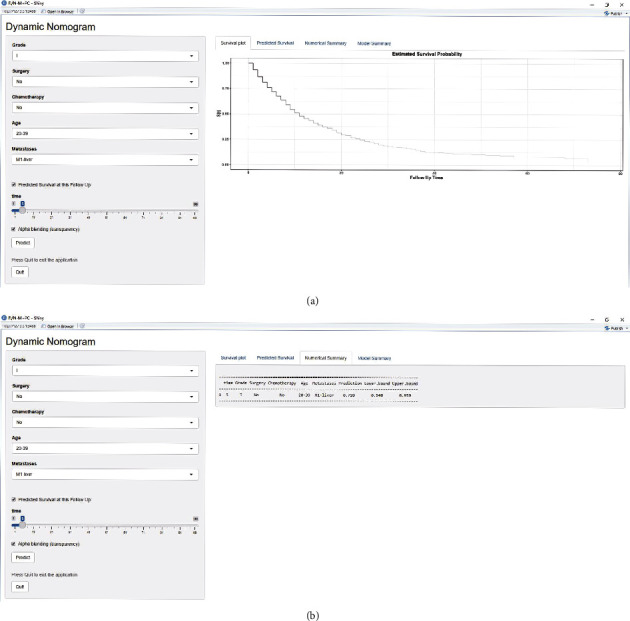
dynamic nomogram prediction model. Fill in the clinicopathological characteristics of the patients, including grade, surgery, chemotherapy, age, and metastases, select the follow-up time and click “Predict” in the dynamic nomogram. Predict the survival rate of patients with the negative lymph node and distant metastasis in different follow-up periods through observing the output pictures and tables, including survival plot, predicted survival, numerical summary, and model summary.

We established and validated the nomogram based on Kaplan–Meier survival curves to demonstrate the prognostic impact of various risk factors on PDAC with *N*0 and distant metastasis patients. Although the corresponding visualization results could be obtained through nomograms, they still lack accuracy and convenience in the clinical application process. Therefore, based on the nomogram prediction results, we established an online nomogram analysis program (the dynamic nomogram calculator) https://lixj3.shinyapps.io/N0M1-PC-DynNomapp/. This online prediction model can be used to selectively input personalized clinical information of patients to predict the survival of individual patients. Compared with the nomogram model, the dynamic nomogram calculator can more conveniently and intuitively predict the survival probability of patients with different clinical and pathological factors at different follow-up time points (Figures 6(a) and 6(b)).

## 4. Discussion

### 4.1. Is There a Definite Association between Regional Lymph Node Metastasis and Distant Metastasis?

Some scholars believe that the steps of tumor cell metastasis to distant sites are orderly and mechanistic. Through dissolution of the extracellular matrix (ECM) and basement membrane, tumor cells enter adjacent tissues, invade lymphatic vessels, and invade blood and distant organs [16]. Regional lymph node metastasis may be the intermediate stage (“springboard”) during the development of distant metastasis. However, this is not absolute, as *N*0 does not mean the absence of distant metastasis. A considerable number of patients with pancreatic cancer will skip the step of regional lymph node metastasis and directly develop distant metastasis [17]. Durczynski et al. [18] also confirmed that cancer in regional lymph nodes can metastasize to distant sites through “jumping,” and thus, the process of distant metastasis is unpredictable. Blood metastasis is also an important pathway that leads to distant metastasis. Tumor cells can directly metastasize to distant organs through the blood without passing through the lymphatic circulation [19, 20]. Ueberroth al. [21] and Yin et al. [22] found that circulating tumor cells (CTCs) and circulating tumor DNA (ctDNA) could be detected in the blood of *N*0 pancreatic cancer patients who achieved pathological complete remission after radical resection of pancreatic cancer or neoadjuvant therapy, which suggests a poor prognosis and a high risk of distant metastasis [23]. Similarly, our study found that among the 4491 PDAC with *N*0 and distant metastasis patients, 1538 (34.24%) also had distant metastasis, which means that the risk of recurrence and metastasis in *N*0 patients may have been previously underestimated. Therefore, it is crucial to predict the risk of distant metastasis in *N*0 patients, and a more appropriate prediction may be an important factor in the improvement inpatient prognosis.

In 1889, Stephen Paget proposed in the “seed and soil hypothesis” that distant metastasis of pancreatic cancer is organ specific [24]. The studies by Shi et al. [25] and Wright et al. [26] have shown that 50% of pancreatic cancers can metastasize to the liver through the portal vein and that the probability of liver metastasis in patients with stage IV pancreatic cancer is as high as 70%. Our study found that among the 4491 patients in PDAC with *N*0, 1538 patients (34.24%) had distant metastasis, of whom 999 (65%) had liver metastasis, which was the most common metastatic site. This finding is consistent with the results of the current study.

### 4.2. What Are the Risk Factors of Distant Metastasis?

Our study found that sex, age, pathological grade, surgery, radiation, race, tumor location, and tumor size were significantly correlated with the development of distant metastasis in PDAC patients. Therefore, we combined these eight clinicopathological factors in a multivariate logistic analysis to predict the risk of distant metastasis in PDAC with *N*0 patients (AUC = 0.87). The results showed that patients with poorly differentiated tumors, those with tumors located in the pancreatic tail, and those with tumors >40 mm were more likely to develop distant metastasis. Multiple studies [27–30] have shown that the location of pancreatic tumors is an important prognostic factor. However, controversy still exists as to which location is associated with a better prognosis. For example, Brennan et al. [31] and Lau et al. [27] reported that patients with tumors located in the pancreatic head were more prone to distant metastasis, which was associated with a poor prognosis. In contrast, studies by Watanabe et al., Artinyan et al., [28, 29], and Ng et al. [32] reported that patients with tumors located in the pancreatic body/tail were more likely to have distant metastasis at the time of treatment and therefore had poor overall survival (OS). The results of our study showed that in PDAC with *N*0 patients, the risk of distant metastasis for tumors in the pancreatic body/tail is higher, and specifically, the risk of developing distant metastasis is 2–2.5 times higher in the pancreatic tail (OR = 4.78, 95% CI 3.71–6.19) than in other sites. This may be because patients with pancreatic head lesions are more prone to biliary obstruction and are diagnosed and treated early due to early symptoms such as jaundice. In contrast, pancreatic body/tail cancer is not easily detected at an early stage and is often diagnosed at a later stage than pancreatic head cancer, which results in a lower surgical resection rate and a worse prognosis [28, 33]. In addition, studies have shown that pancreatic body/tail lesions exhibit more invasive tumor biological characteristics [34], which may also be the reason why pancreatic body/tail tumors are prone to distant metastasis and a poor prognosis.

In addition, we found that older age and smaller tumor size (21–40 mm) were associated with a lower risk of developing distant metastasis. Particularly for patients aged ≥80 years, the risk of distant metastasis was almost 2.5–3 times lower than that of patients aged 60–79 and 40–59 years (OR: 0.36 vs. 0.8 vs. 1.01). Although we found that age ≥80 years is a protective factor for distant metastasis in pancreatic cancer, considering the decline in various physiological functions and performance statuses in elderly patients, treatment tolerance is poor, which seriously affects the prognosis of elderly patients [35, 36]. Therefore, although the risk of distant metastasis in the elderly population is lower than that in the younger population, the long-term prognosis of the younger population is better than that of the elderly population [37]. Michelakos et al. [38] found that the PDAC tumor size larger than 25 mm was an independent predictor of decreased OS (HR = 1.7, *P*=0.03). This may be because larger tumors are more likely to invade the surrounding tissues of the pancreas and the peripheral nerve plexus. The more extensive the lymph node metastasis and local micrometastasis are, the more likely distant metastasis is to occur and the worse the prognosis is [39]. The oxygenation of tumors in the rodent tumor model KHT-C decreased as the tumor volume decreased, and it was found that hypoxic tumors were more prone to distant metastasis, which suggests that the hypoxic environment caused by changes in the tumor size may be related to the mechanism of metastasis of human tumors [40].

We found that patients with PDAC who had undergone surgery (OR = 0.05, 95% CI: 0.03–0.06, *P* < 0.001) or who had received radiation therapy (OR = 0.15, 95% CI: 0.12–0.19, *P* < 0.001) had a lower risk of distant metastasis. Generally, patients with advanced pancreatic cancer have lost the opportunity for surgical treatment. However, with the recent application and maturation of minimally invasive techniques, the use of palliative surgery for the treatment of advanced pancreatic cancer under the premise of strict control of indications has been widely accepted by clinicians [41–43]. Radiation therapy is also an important local treatment modality for pancreatic cancer. Radiation may be performed at all stages and is often used as postoperative adjuvant radiotherapy, locally advanced palliative radiotherapy, and intraoperative radiotherapy [44, 45]. However, the role and status of radiotherapy in the treatment of pancreatic cancer are not fully understood. In summary, palliative surgery and radiotherapy can reduce distant metastasis, relieve pain symptoms, improve quality of life, and improve overall efficacy. Our findings support this conclusion. However, the timing, type, and scope of surgery as well as the method, dose, and scope of radiotherapy are still controversial [46–50].

### 4.3. The Nomogram Model and Online Dynamic Calculator for the 1-, 2-, and 3-Year CSS

In this study, age, pathological grade, surgery, chemotherapy, and metastatic site were identified as predictors of 1-, 2-, and 3-year CSS in PDAC with *N*0 and distant metastasis patients. The results of the multivariate Cox analysis suggested that the 1-, 2-, and 3-year CSS was lower in patients with distant metastasis who were older, those with more poorly differentiated tumors, and those with multiple metastases. In contrast, treatment with surgery and chemotherapy resulted in higher 1-, 2-, and 3- year CSS. Li et al. [51] divided patients with pancreatic cancer with liver metastasis into low-, medium-, and high-risk groups. The results showed that the CSS of patients in the medium- and high-risk groups who were treated with surgery, chemotherapy, and radiotherapy was significantly higher than the CSS of those who did not receive these treatments. Macchini et al. [52] found that the prognosis of elderly patients with pancreatic cancer is usually worse than that of younger patients, which may be due to the poor physical condition and more treatment-related complications in elderly patients. Vincent et al. [53] believed that the degree of differentiation is a key factor for the prognosis of PDAC, and poor differentiation indicates poor prognosis. The conclusions of the abovementioned studies are consistent with our findings.

Subsequently, we constructed and validated the nomogram prediction model. The *C*-index and AUC were used to assess the accuracy and discrimination ability of the nomogram in PDAC with *N*0 and distant metastasis patients. The results showed that the *C*-index values of the training and the validation cohorts were 0.7 and 0.69, respectively. The AUCs for the 1-, 2-, and 3-year CSS were 0.76, 0.77, and 0.71 and 0.76, 0.69, and 0.79, for the training and validation cohorts, respectively. The prediction data of this nomogram are highly consistent with the actual data, which indicates good prediction ability. The calibration curves showed that the predicted value and the actual value were highly coincident and that there was good consistency, which demonstrates that the prediction performance of the model is good. The TNM staging system (AJCC 7th edition) has been widely accepted as an important reference standard for the pancreatic cancer assessment and can provide a basis for developing treatment plans and assessing prognosis. The DCA curves showed that the nomogram had a good predictive ability for the 1-, 2-, and 3-year CSS in PDAC with *N*0 and distant metastasis patients, and the predictive power was significantly better than that of the traditional AJCC TNM staging system. In addition, the patients were divided into three groups: low-, medium-, and high-risk. The results showed that the median CSS and the 1-, 2-, and 3-year CSS of the patients were correspondingly reduced for each elevated risk level, which suggests that the lower the risk grouping is, the higher the survival rate is.

Nomograms are currently one of the main types of clinical prediction models. Although nomograms have achieved the visualization of predictive models to a large extent, we admit that their practical application has many shortcomings, especially in today's era of precision medicine. On the one hand nomograms are not accurate and are not easy to use, and on the other hand, a nomogram cannot display the prediction results of a nonlinear model. Therefore, we also designed the online dynamic nomogram calculator for predicting the survival rate of PDAC patients with *N*0 and distant metastasis, which completely overcomes the abovementioned shortcomings.

### 4.4. Limitations of the Study

Our study also had some limitations. First, this was a retrospective study based on the SEER database. Considering that the information of the important prognostic factors of some cases in the SEER database was incomplete (such as a lack of detailed chemotherapy regimens and doses), there might b e a certain degree of bias. Second, this study was a single retrospective analysis, and thus, prospective and multicenter studies with larger sample sizes are needed for validation to make the model more convincing.

## 5. Conclusions

Pathological grade, tumor location, and tumor size were independent risk factors for distant metastasis in PDAC with *N*0. Older age, smaller tumor size, surgery, and radiotherapy were protective factors against distant metastasis. The nomogram we constructed could effectively predict CSS in PDAC with *N*0 and distant metastasis and improve the classification of the risk. Furthermore, an online dynamic nomogram calculator was established.

## Figures and Tables

**Table 1:** Clinicopathological characteristics of PDAC patients with the negative lymph node status but distant metastasis in the training and validation cohorts.

	Levels	Overall cohort	Training cohort	Validation cohort	*P* value
*n*		1538	1083	455	

Sex	Female	738 (48.0)	523 (48.3)	215 (47.3)	0.752
	Male	800 (52.0)	560 (51.7)	240 (52.7)	

Age (years)	20–39	10 (0.7)	8 (0.7)	2 (0.4)	0.75
	40–59	405 (26.3)	279 (25.8)	126 (27.7)	
	60–79	936 (60.9)	666 (61.5)	270 (59.3)	
	≥80	187 (12.2)	130 (12.0)	57 (12.5)	

Pathological grade	I	120 (7.8)	86 (7.9)	34 (7.5)	0.951
	II	638 (41.5)	449 (41.5)	189 (41.5)	
	III/IV	780 (50.7)	548 (50.6)	232 (51.0)	

Surgery	No	1450 (94.3)	1020 (94.2)	430 (94.5)	0.898
	Yes	88 (5.7)	63 (5.8)	25 (5.5)	

Radiation	No	1442 (93.8)	1023 (94.5)	419 (92.1)	0.101
	Yes	96 (6.2)	60 (5.5)	36 (7.9)	

Chemotherapy	No	510 (33.2)	348 (32.1)	162 (35.6)	0.207
	Yes	1028 (66.8)	735 (67.9)	293 (64.4)	

Race	Black	227 (14.8)	147 (13.6)	80 (17.6)	0.122
	Other^a^	114 (7.4)	83 (7.7)	31 (6.8)	
	White	1197 (77.8)	853 (78.8)	344 (75.6)	

Location	Head	607 (39.5)	439 (40.5)	168 (36.9)	0.167
	Body	268 (17.4)	185 (17.1)	83 (18.2)	
	Tail	324 (21.1)	235 (21.7)	89 (19.6)	
	Other^b^	339 (22.0)	224 (20.7)	115 (25.3)	

Tumor size (mm)	0–20	84 (5.5)	61 (5.6)	23 (5.1)	0.848
	21–40	579 (37.6)	404 (37.3)	175 (38.5)	
	>40	875 (56.9)	618 (57.1)	257 (56.5)	

Metastasis	*M*1-liver	999 (65.0)	710 (65.6)	289 (63.5)	0.899
	*M*1-lung	97 (6.3)	67 (6.2)	30 (6.6)	
	*M*1-other^c^	270 (17.6)	187 (17.3)	83 (18.2)	
	*M*1-multiple	172 (11.2)	119 (11.0)	53 (11.6)	

Pathological grade: I, well differentiated; II, moderately differentiated; III/IV, poorly differentiated and undifferentiated. Other^a^: defined as the Asian/Pacific Islander and American Indian/Alaska native; other^b^: the neck and multiple; other^c^: metastasis to other sites except the liver, the lung and multiple sites; *M*1: distant metastasis; multiple: two or more distant metastasis sites.

**Table 2:** Univariate and multivariate logistic regression analyses of clinical variables correlated with distant metastasis in PDAC with the negative lymph node status.

Variables	Subgroups	Univariable		Multivariable	
		OR	*P* value	OR	*P* value
Sex	Female	Reference	0.002	Reference	0.528
	Male	1.22 (1.08–1.38)		1.03 (0.88–1.21)	

Age (years)	20–39	Reference	**<0.001**	Reference	**<0.001**
	40–59	1.46 (0.71–3.23)		1.01 (0.39–2.65)	
	60–79	1.25 (0.61–2.76)		0.8 (0.32–2.1)	
	≥80	0.95 (0.46–2.12)		0.36 (0.14–0.94)	

Pathological grade	I	Reference	**<0.001**	Reference	**<0.001**
	II	1.68 (1.35–2.1)		1.77 (1.35–2.32)	
	III/IV	2.59 (2.08–3.24)		2.05 (1.57–2.68)	

Surgery	No	Reference	**<0.001**	Reference	**<0.001**
	Yes	0.05 (0.04–0.06)		0.05 (0.03–0.06)	

Radiation	No	Reference	**<0.001**	Reference	**<0.001**
	Yes	0.14 (0.11–0.17)		0.15 (0.12–0.19)	
Chemotherapy	No	Reference	0.941	—	
	Yes	1 (0.87–1.13)			

Race	White	Reference	**0.005**	Reference	0.220
	Black	1.2 (1–1.43)		0.92 (0.73–1.15)	
	Other^a^	0.76 (0.61–0.96)		0.79 (0.59–1.04)	

Location	Head	Reference	**<0.001**	Reference	**<0.001**
	Body	2.5 (2.08–3.01)		2.25 (1.79–2.84)	
	Tail	3.9 (3.24–4.7)		4.78 (3.71–6.19)	
	Other^b^	2.72 (2.29–3.23)		1.87 (1.52–2.31)	

Tumor size (mm)	0–20	Reference	**<0.001**	Reference	**0.006**
	21–40	2 (1.57–2.59)		0.95 (0.69–1.3)	
	>40	4.74 (3.71–6.12)		1.24 (0.9–1.71)	

Other^a^: defined as the Asian/Pacific Islander and American Indian/Alaska native; other^b^: the neck and multiple. PDAC: pancreatic ductal adenocarcinoma; pathological grade: I, well differentiated; II, moderately differentiated; III/IV, poorly differentiated and undifferentiated; OR: odds ratio. Bold values indicate statistical significance (*P* < 0.05).

**Table 3:** Univariate and multivariate cox regression analyses of predictive variables correlated with CSS in lymph-node-negative PDAC with distant metastasis.

Variables	Subgroups	Univariable		Multivariable	
		HR	*P* value	HR	*P* value
Sex	Female	Reference	0.672	—	
	Male	1.03 (0.91–1.16)			

Age (years)	20–39	Reference	**<0.001**	Reference	**<0.001**
	40–59	2.39 (1.06–5.36)		2.23 (0.99–5.02)	
	60–79	2.74 (1.23–6.13)		2.37 (1.06–5.32)	
	≥80	4.79 (2.11–10.89)		3.69 (1.62–8.43)	

Pathological grade	I	Reference	**<0.001**	Reference	**<0.001**
	II	1.41 (1.11–1.8)		1.5 (1.18–1.91)	
	III/IV	1.97 (1.55–2.49)		2.02 (1.58–2.57)	

Surgery	No	Reference	**<0.001**	Reference	**<0.001**
	Yes	0.45 (0.34–0.59)		0.45 (0.34–0.59)	

Radiation	No	Reference	0.05	—	
	Yes	0.78 (0.6–1.01)			

Chemotherapy	No	Reference	**<0.001**	Reference	**<0.001**
	Yes	0.44 (0.39–0.51)		0.45 (0.39–0.52)	

Race	White	Reference	0.102	—	
	Black	1.19 (0.99–1.41)			
	Other^a^	0.9 (0.72–1.14)			

Location	Head	Reference	0.2	—	
	Body	0.96 (0.81–1.15)			
	Tail	1.04 (0.88–1.22)			
	Other^b^	1.17 (1–1.38)			

Tumor size (mm)	0–20	Reference	0.192	—	
	21–40	1.2 (0.91–1.58)			
	>40	1.26 (0.97–1.65)			

Metastasis	*M*1-liver	Reference	**<0.001**	Reference	**<0.001**
	*M*1-lung	0.84 (0.65–1.08)		0.81 (0.62–1.05)	
	*M*1-other^c^	0.73 (0.62–0.86)		0.8 (0.68–0.95)	
	*M*1-multiple	1.29 (1.06–1.57)		1.31 (1.07–1.59)	

Other^a^: defined as the Asian/Pacific Islander and American Indian/Alaska native; other^b^: the neck and multiple; other^c^: metastasis to other sites except the liver, the lung and multiple sites; pathological grade: I, well differentiated; II, moderately differentiated; III/IV, poorly differentiated and undifferentiated; M1: distant metastasis; multiple: two or more distant metastasis sites; CSS: cancer-specific survival; PDAC: pancreatic ductal adenocarcinoma; HR: hazard ratio; bold values indicate statistical significance (*p* < 0.05).

**Table 4:** The specific value of clinicopathological factors in the nomogram in the training cohort.

Characteristics	Scores
Pathological grade	
I	0
II	31
III/IV	54
Surgery	
No	61
Yes	0
Chemotherapy	
No	61
Yes	0
Age	
20–39	0
40–59	61
60–79	66
≥80	100
Metastasis	
*M*1-liver	17
*M*1-lung	1
*M*1-other	0
*M*1-multiple	37
Total point for 1-year CSS	
0.1	221
0.2	194
0.3	171
0.4	150
0.5	129
0.6	106
0.7	78
Total point for 2-year CSS	
0.1	177
0.2	149
0.3	127
0.4	106
0.5	85
0.6	61
Total point for 3-year CSS	
0.1	152
0.2	125
0.3	103
0.4	82
0.5	60

Pathological grade: I, well differentiated; II, moderately differentiated; III/IV, poorly differentiated and undifferentiated; other: metastasis to other sites except the liver, the lung, and multiple lesions. CSS: cancer-specific survival; *M*1: distant metastasis; multiple: two or more distant metastasis sites.

## Data Availability

All data generated or analyzed during this study are included within the article.

## Supplementary Materials

Supplementary Materials Figure S1: The receiver operating characteristics (ROC) curve and area under the ROC curve (AUC) for the logistics regression model. The AUC value for the logistics regression model was 0.87. Figure S2: The median CSS and 1-, 3-, and 5-year CSS of the overall cohort (A), the training cohort (B), and the validation cohort (C) in PDAC with *N*0 and distant metastasis patients. Figure S3(A)–(C) Calibration curves of the validation cohort for evaluating the accuracy of the nomogram. (A) 1-year CSS in lymph-node-negative PDAC with distant metastasis, (B) 2-year CSS in lymph-node-negative PDAC with distant metastasis, and (C) 3-year CSS in lymph-node-negative PDAC with distant metastasis. The solid green line represents the performance of the nomogram, of which the closer fit to the dotted black line represents the better prediction of the nomogram we constructed. Table S1: The Kaplan–Meier survival curves for predicting the CSS of lymph-node-negative PDAC with distant metastasis. Table S2: The 1-, 2-, and 3-year AUC values and the C-index in the training cohort and validation cohort.
