# Supplementary material for: Analysis of Risk Factors for Distant Metastasis of Pancreatic Ductal Adenocarcinoma without Regional Lymph Node Metastasis and a Nomogram Prediction Model for Survival
Source: Evid Based Complement Alternat Med. 2023 Feb 21;2023:2916974. doi: 10.1155/2023/2916974 (PMC9974279; doi:10.1155/2023/2916974)
Supplement: Supplementary Materials — Figure S1: The receiver operating characteristics (ROC) curve and area under the ROC curve (AUC) for the logistics regression model. The AUC value for the logistics regression model was 0.87. Figure S2: The median CSS and 1-, 3-, and 5-year CSS of the overall cohort (A), the training cohort (B), and the validation cohort (C) in PDAC with N0 and distant metastasis patients. Figure S3(A)–(C) Calibration curves of the validation cohort for evaluating the accuracy of the nomogram. (A) 1-year CSS in lymph-node-negative PDAC with distant metastasis, (B) 2-year CSS in lymph-node-negative PDAC with distant metastasis, and (C) 3-year CSS in lymph-node-negative PDAC with distant metastasis. The solid green line represents the performance of the nomogram, of which the closer fit to the dotted black line represents the better prediction of the nomogram we constructed. Table S1: The Kaplan–Meier survival curves for predicting the CSS of lymph-node-negative PDAC with distant metastasis. Table S2: The 1-, 2-, and 3-year AUC values and the C-index in the training cohort and validation cohort. [file 2916974.f1.zip › Table S1 (1).docx]

**Table S1.** The Kaplan–Meier survival curves for predicting the CSS of lymph-node-negative PDAC with distant metastasis.

| **Subgroup** | **median CSS (month)** | **1-year CSS (%)** | **2-year CSS (%)** | **3-year CSS (%)** |
| --- | --- | --- | --- | --- |
| **Pathological grade** |  |  |  |  |
| I | 9.0 (7.0-14.0) | 38.9 (29.8-50.8) | 18.9 (12.1-29.3) | 6.5 (2.9-14.9) |
| II | 6.0 (6.0-7.0) | 25.2 (21.5-30.0) | 9.6 (7.2-12.7) | 4.7 (3.1-7.2) |
| III/IV | 4.0 (4.0-5.0) | 15.1 (12.3-19.0) | 4.4 (3.0-6.5) | 1.4 (0.7-2.8) |
| **Sugery** |  |  |  |  |
| No | 5.0 (4.0-6.0) | 19.5 (17.2-22.1) | 6.2 (4.9-7.9) | 2.3 (1.5-3.5) |
| Yes | 11.0 (8.0-20.0) | 47.6 (36.8-61.7) | 28.6 (19.3-42.2) | 15.4 (8.6-27.7) |
| **Chemotherapy** |  |  |  |  |
| No | 2.0 (2.0-2.0) | 7.5 (5.2-10.9) | 3.1 (1.7-5.7) | 2.5 (1.3-4.9) |
| Yes | 7.0 (7.0-8.0) | 27.6 (24.5-31.0) | 9.7 (7.8-12.1) | 3.5 (2.4-5.2) |
| **Age (Years)** |  |  |  |  |
| 20-39 | 16.0 (12.0-NR) | 62.5 (36.5-100.0) | 25.0 (7.5-83.0) | NR |
| 40-59 | 7.0 (6.0-8.0) | 26.7 (22.0-32.4) | 10.5 (7.4-14.8) | 3.9 (2.2-7.0) |
| 60-79 | 5.0 (5.0-6.0) | 21.3 (18.4-24.7) | 7.2 (5.5-9.5) | 3.0 (1.9-4.7) |
| ≥80 | 3.0 (2.0-4.0) | 6.2 (3.1-12.0) | 2.3 (0.8-7.1) | 1.5 (0.4-6.1) |
| **Metastasis** |  |  |  |  |
| M1-liver | 5.0 (4.0-6.0) | 20.4 (17.7-23.7) | 6.9 (5.3-9.1) | 2.9 (1.9-4.4) |
| M1-lung | 6.0 (5.0-8.0) | 22.4 (14.3-35.0) | 10.5 (5.2-21.1) | 1.5 (0.2-10.4) |
| M1-other | 8.0 (6.0-9.0) | 31.0 (25.0-38.4) | 11.8 (7.9-17.4) | 5.4 (2.9-10.0) |
| M1-multiple | 4.0 (3.0-5.0) | 9.2 (5.3-16.2) | 3.4 (1.3-8.8) | NR |
| **Risk group** |  |  |  |  |
| Low risk (≤175) | 11.0 (9.0-13.0) | 45.0 (39.0-51.9) | 19.1 (14.6-24.9) | 6.8 (4.2-11.1) |
| Median risk (>175 & ≤231) | 6.0 (6.0-7.0) | 19.9 (16.8-23.4) | 6.3 (4.5-8.7) | 2.2 (1.3-3.9) |
| High risk (>231) | 2.0 (2.0-2.0) | 5.3 (3.3-8.5) | 1.5 (0.6-3.9) | 1.1 (0.4-3.4) |

**Abbreviations:** CSS: cancer-specific survival; PDAC: pancreatic ductal adenocarcinoma; Pathological grade: I, well differentiated; II, moderately differentiated; III/IV, poorly differentiated and undifferentiated; M1: distant metastasis; Other: metastasis to other sites except liver, lung and multiple.
